# Supervisor bottom line mentality and its impact on employee outcomes: The mediating role of employee appraisals

**DOI:** 10.1371/journal.pone.0338024

**Published:** 2026-01-20

**Authors:** Zainab Naseer, Sayyed Muhammad Mehdi Raza Naqvi

**Affiliations:** Department of Management and Social Sciences, Capital University of Science and Technology, Islamabad, Pakistan; IUB: The Islamia University of Bahawalpur Pakistan, PAKISTAN

## Abstract

The concept of Bottom-line mentality (BLM) has been increasingly emphasized in contemporary workplaces, often at the expense of other critical organizational priorities. However, its implications for employees have remained underexplored. This study, which investigates how supervisors’ BLM influences employees’ behaviors and perceptions in the workplace, underscores the need for a balanced approach to leadership. Using SmartPLS 4, survey data from 382 employees in Pakistan’s fast-moving consumer goods sector were analyzed. The results reveal that supervisor BLM is positively associated with employee incivility and negatively associated with employees’ goal progress. Furthermore, employees’ hindrance appraisals mediate the relationship between supervisor BLM and incivility, whereas challenge appraisals mediate the relationship between supervisor BLM and goal progress. This research extends the literature by clarifying how supervisors’ bottom-line focus shapes employee actions and work outcomes, and it highlights the importance of a balanced leadership approach. The findings suggest that although leaders may adopt a bottom-line mentality to enhance performance, its effectiveness depends on how employees interpret and respond. The study offers practical guidance for organizations seeking to strengthen feedback systems and reduce the negative effects of supervisory misconduct.

## Introduction

In today’s highly competitive business environment, organizations increasingly prioritize a bottom-line mentality (BLM), that is, a focus on monetary outcomes, such as profits, as the primary or even sole indicator of success [1–3]. Although it is often considered a necessity for the survival of an organization, especially when it is under economic pressure [4], it can lead to neglecting other vitally important values and processes. Although the term “bottom line mentality” is new, the idea of focusing on financial outcomes more than on anything else has long shaped managerial practice [5]. As [6] suggests, the problem is not that organizations seek to pursue financial goals but that they often neglect other goals in doing so.

Empirical research on BLM has produced mixed findings, suggesting it may function as a double-edged sword [7]. Supporting a beneficial relationship between the employee and the organization, some studies point towards possible benefits (e.g., improved task and customer performance). However, there is accumulating evidence of negative outcomes (reduced organizational citizenship behaviors) [8,9], diminished overall performance [10], and increased workplace conflict [9]. These conflicting results underscore the need to examine when and why BLM produces positive versus negative outcomes within organizations.

Despite growing recognition of the impact of supervisor bottom-line mentality (SBLM), significant theoretical gaps remain. Specifically, prior research has largely overlooked how employees’ subjective interpretations of SBLM, whether they perceive it as a challenge or a hindrance, influence their reactions and workplace outcomes. Yet, much of the existing literature makes the extreme assumption that SBLM is either unanimously bad or good for employees or ignores the mediating psychological processes that translate SBLM into employee responses [11–13]. As a result, little is known about the mechanisms through which SBLM exerts its effects and under what conditions its outcomes are adaptive or maladaptive. This study addresses this weakness by articulating the research gap more sharply, emphasizing the novelty of examining appraisals as a mediating lens to explain how SBLM influences employee behavior.

In an attempt to address these gaps, the present study adopts the Transactional Theory of Stress and Coping (TTSC) [14] as a conceptual framework to examine how SBLM affects key workplace outcomes, mediated by employees’ appraisals of SBLM. Based on the information provided by TTSC, employees undergo a two-step appraisal process regarding workplace stressors. The primary appraisal involves evaluating whether a stressor intimidates or challenges them, while the secondary appraisal assesses their ability to deal with it. This framework suggests that individual differences in appraisal are central to understanding how SBLM is experienced and enacted in the workplace. For instance, one employee may interpret a supervisor’s strong focus on the bottom line as motivating and energizing, while another may see it as threatening and harmful.

Pakistan provides an important setting for examining supervisor bottom line mentality because cultural norms fundamentally shape how employees interpret and respond to leader behaviors. Pakistani workplaces are characterized by hierarchical relationships, collectivist values and strong dependence on supervisors for evaluations, opportunities and informal support. Such cultural features intensify the psychological impact of the supervisor bottom line focus, making employees more likely to tolerate pressure and voice concerns. Such dynamics can change the appraisal processes described in transactional theory of stress and coping, which means that same bottom line behavior may be experienced differently than in western samples. Studying SBLM in Pakistan therefore does more than extend the existing research to a new context, i.e., it provides a theoretically meaningful test of whether appraisal based mechanisms generalize across cultures or function differently when authority, dependence and performance pressure are more prominent.

This study makes several contributions to the literature. First, it focuses on employees’ interpretations of SBLM, thereby offering insight into the subjective mechanisms behind different reactions in the workplace. Second, the study examines challenge and hindrance appraisals to clarify the inconsistent effects found in previous studies. Third, it highlights both the mediating role of appraisals and the Pakistani cultural setting as unique contributions, providing a stronger foundation for the study’s novelty. Ultimately, these insights have practical significance for organizational leaders, who can utilize them to understand the extent to which employees internalize bottom-line priorities and the potential consequences for both performance and well-being.

## Hypothesis development

### Supervisor bottom-line mentality and employee appraisal

Bottom-line mentality (BLM) is characterized by a one-dimensional focus on achieving financial outcomes, often at the expense of other critical organizational priorities [13]. In practical terms, this manifests in performance expectations closely tied to productivity and profitability. While the pursuit of monetary success is not inherently problematic, it becomes concerning when leaders’ single-minded emphasis on financial results leads to the neglect of employee well-being, ethical standards, and long-term organizational health [15].

Management strategies have varying effects across organizations. According to the Transactional Theory of Stress and Coping, people at work do not only react to stressors but also take time to evaluate and assess them through a two-step method. The first step involves determining whether a specific stressor is perceived as a source of opportunities for improvement and success or as something blocking, dangerous, or harmful [7]. Challenge appraisals occur in situations where individuals perceive workplace demands as challenging (i.e., motivating and potentially skill-developing, recognition-giving, or advancement-conferring). Conversely, hindrance appraisals refer to the assumption that there is a chance these demands may cause frustration, restriction, or loss that thereby pose obstacles to progress or livelihood [16].

Research suggests that these appraisals, rather than the objective demands themselves, largely determine employees’ reactions and subsequent outcomes [17,18]. When employees interpret a supervisor’s bottom-line focus as a challenge, they may feel motivated to meet ambitious goals, which can enhance their performance. Conversely, if the same bottom-line emphasis is seen as a hindrance, it may evoke stress, reduce engagement, and foster counterproductive behaviors [19].

However, the literature reveals a lack of consensus regarding how employees assess their supervisor’s bottom-line mentality (SBLM). Some research suggests that SBLM enhances output under certain conditions. At the same time, other findings suggest it can lead to less helpful behavior at work and increased anxiety and unhappiness at the job [8–10]. This inconsistency suggests that individual differences in appraisal play a crucial mediating role [20].

Prior BLM research has largely linked SBLM to unethical outcomes through moral disengagement pathways. TTSC has rarely been used to specify how employees convert leader priorities into distinct responses. This study integrates TTSC to argue that SBLM is a stressor that splits into two appraisal routes, i.e., hindrance, which fuels strain and incivility, and challenge, which supports goal progress. This dual route mechanism explains positive and negative findings within one framework by tying them to how employees evaluate the same supervisory pressure.

Accordingly, we hypothesize the following:

**Hypothesis 1a:** Supervisor bottom-line mentality (SBLM) is positively associated with employee hindrance appraisal.

**Hypothesis 1b:** Supervisor bottom-line mentality (SBLM) is positively associated with employee challenge appraisal.

### SBLM and employee incivility

Supervisors who strongly endorse a bottom-line mentality (SBLM) may unintentionally create a high-pressure work atmosphere where employees feel unimportant or neglected [21,22]. Previous research suggests that environments primarily focused on financial gain often overlook the emotional well-being and interpersonal dynamics of employees [23]. This type of air in the workplace over the long term may increase workers’ stress and make it difficult for them to manage their emotions and treat each other with respect [24].

Incivility, defined as low-intensity deviant behavior with ambiguous intent to harm [25], is particularly likely to flourish in contexts characterized by stress, ambiguity, and diminished respect [26]. Many studies have shown that when rules for respecting people are not established, there is a greater likelihood of subtle mistreatment in the workplace [26,27]. A leader’s actions are crucial in shaping a workplace climate. Putting financial benefits in front of everything else may lead supervisors to neglect the importance of caring for employees and supporting teamwork [28]. Based on the Transactional Theory of Stress and Coping, employees may exhibit signs of incivility toward leaders to push back or ease tension when they perceive their leadership as not providing them with sufficient attention [29].

Drawing on the Transactional Theory of Stress and Coping [14], current study argues that SBLM functions as a situational stressor that employees must appraise and respond to. Clearly, when supervisors emphasize financial outcomes over social or relational concerns, employees may appraise this as a hindrance stressor that hinders their need for recognition and respect [30]. Similar appraisals increase the likelihood of negative emotional responses, which, in turn, may manifest as uncivil behaviors. This TTSC-based framing moves beyond prior BLM research, which has primarily emphasized leader priorities and organizational culture, by emphasizing employees’ appraisal processes and coping responses as the key and fundamental mechanism linking SBLM to incivility.

TTSC offers a unique lens that integrates the leader-driven emphasis on bottom-line outcomes with employees’ psychological reactions. This research, departing from earlier work on BLM, positions BLM not only as a contextual factor but also as a stressor that directly shapes and shapes employee coping strategies. This deviation is a significant contribution to the field.

In addition, employees affected by SBLM-style leadership may notice that supervisors prioritize achieving results over monitoring employee behavior, which can lead to a sense of less responsibility for their civil conduct [24]. If an organization fails to provide adequate emotional support, employees are likely to behave disrespectfully, even if this is not their intention.

Drawing on these insights and integrating theoretical and empirical evidence, the following hypothesis is proposed:

**Hypothesis 2**: SBLM is positively related to employee incivility

### SBLM and employee goal progress

Employee goal progress refers to the ongoing advancement employees make towards fulfilling work-related and personal objectives, serving as a critical indicator of performance, motivation, and organizational effectiveness [31; 32]. Numerous studies suggest that when employees work towards significant goals and notice progress, they report greater satisfaction, increased involvement, and improved success [33,34]. However, the ability to sustain goal progress, especially under stressful conditions, is closely tied to the coping strategies available to employees [35].

Problem-focused coping (e.g., prioritizing tasks, seeking help, and innovating solutions) is associated with better outcomes in high-pressure environments [14]. Yet, not all workplace stressors encourage adaptive coping. When supervisors emphasize a bottom-line mentality (SBLM), that is, placing exclusive focus on financial results to the detriment of other priorities, they may unintentionally undermine employees’ pursuit of personal and professional goals which may hinder their ability to effectively progress towards achieving these goals [31]. The environments lacking resources or support can shift employees toward emotion-focused coping strategies, such as disengagement or denial, which are less effective for goal attainment [36].

By applying TTSC [14], this research convincingly argues that supervisor bottom-line mentality (SBLM) is a potent stressor that influences employees’ appraisal and coping responses. Whenever supervisors prioritize financial outcomes over employee needs, it creates a hindrance stressor [37], which can impede employees’ ability to engage in meaningful attainment of goals [30].

Under such circumstances, employees may shift from problem-focused coping to less effective emotion-focused strategies, such as disengagement or denial [38]. The TTSC-based framework not only builds on previous research on BLM but also provides significant practical insights into how supervisor priorities can shape the workplace climate and how employees’ appraisal and coping mechanisms can directly impact goal progress.

Additionally, SBLM may increase targets at work while reducing support and attention to employees’ feelings and career advancement [22]. This can result in employees feeling that their jobs are less meaningful as the organization takes priority, which leads to their demotivation [24]. Although SBLM can define the main aims of the organization, placing too much stress on reaching financial targets without adequate support for employees can lead to personal goals suffering, especially if they don’t align with the organization’s objectives [35].

In sum, integrating these theoretical and empirical insights highlights a consistent pattern:

**Hypothesis 3:** Supervisor bottom-line mentality (SBLM) is negatively associated with employee goal progress.

### Indirect effects of supervisor bottom-line mentality on employee outcomes through employee appraisals

Drawing upon the preceding literature and hypotheses, we propose a key insight. We suggest that employees’ appraisals are a crucial psychological mechanism, heavily influenced by the supervisor’s bottom-line mentality (SBLM), and in turn, significantly impacting workplace outcomes [14]. This is not a passive reaction. Recent empirical research emphasizes the active role of employees in interpreting their supervisor’s behavior, thereby shaping their emotional and behavioral responses [39]. This highlights the agency of the employees in the workplace [40].

According to TTSC, hindrance appraisal occurs when individuals perceive the pressure from SBLM as obstructive to their goals, viewing organizational expectations as misaligned with personal or professional objectives. Such examinations often result in anger, diminished morale, and disengagement from work responsibilities, which in turn can boost uncivil behaviors such as rudeness, gossiping, or even sabotage [38]. Supporting this view, management research suggests that acknowledging hindrances can lead employees to distance themselves from colleagues and withdraw from work activities psychologically [38,41].

In lieu of this, TTSC also posits that when employees interpret SBLM as an opportunity to grow, acquire skills, or advance in their careers, a challenge appraisal emerges. In this appraisal process, pressure is reframed as motivating, fostering adaptive coping strategies, stronger motivation, and enhanced goal progress [42]. Employees who appraise SBLM as a challenge may anticipate recognition, rewards, or developmental opportunities, accordingly sustaining effort and engagement [42].

Integrating these perspectives highlights that employees’ appraisals of SBLM, assuming that as a hindrance or a challenge, significantly influence its consequences. Hindrance appraisals are more likely to produce negative outcomes, such as incivility, whereas challenge appraisals can facilitate positive outcomes, such as goal progress.

Based on this framework, we propose the following mediation hypotheses

**Hypothesis 4a:** Employee hindrance appraisal mediates the relationship between supervisor bottom-line mentality and employee incivility.

**Hypothesis 4b:** Employee challenge appraisal mediates the relationship between supervisor bottom line mentality and employee goal progress.

### Challenge appraisal as a mediator of the SBLM and incivility relationship

While challenge appraisals are frequently associated with positive employee outcomes, such as heightened motivation, proactive behavior, and engagement [14,42], emerging evidence suggests that these effects may not always be uniformly beneficial, especially in the context of a supervisor’s bottom-line mentality. SBLM, defined as a leadership style characterized by a singular focus on financial outcomes at the expense of other organizational goals [13], can be appraised by employees as a challenge when they perceive performance pressure as an opportunity for advancement or personal growth [17,43]. In such situations, workers may work extremely hard and take significant steps to meet the company’s high expectations.

According to Transactional Theory of Stress and Coping, when employees interpret their supervisor’s bottom line focus as a challenge, they may be less likely to engage in counterproductive behaviors. However, studies have found that how individuals view challenges in high-paced, achievement-focused situations can sometimes lead to unexpected problems [42]. As a result, employees motivated only by achieving their leaders’ high goals may become so eager for results that they neglect their relationships and the usual norms in the workplace [43]. If workers believe their supervisors focus mainly on work completion, they may be less sensitive to social rules and show more uncivil behavior. Hence, by putting all these facts together, it is reasonable to conclude that the relationship between SBLM and workplace incivility involves challenge appraisal as a mediator: SBLM can encourage employees to interpret issues as challenges, but in that process, it can lead them to focus on completing tasks at the cost of maintaining good relationships, which might result in more workplace incivility [41].

**Hypothesis 5a:** Employee challenge appraisal mediates the relationship between supervisor bottom-line mentality and employee incivility.

### Hindrance appraisal as a mediator of the SBLM–goal progress relationship

Employees working under supervisors with sole focus on financial outcomes develop a high-pressure work environment where they lack support for their professional development [13]. Employees go further than just responding; they also judge how supervisors behave. If it is perceived as blocking their growth and out of line, it becomes a hindrance [30,14].

Hindrance appraisal means that people at work view barriers as hindrances to their success in achieving goals, which reduces their progress in both personal and professional areas. According to Transactional Theory of Stress and Coping, hindrance appraisals may lead individuals to suffer from reduced motivation and poor mental states [42]. If employees perceive their supervisor’s behavior negatively, they may feel less capable of achieving their goals. This can trigger disengagement or avoidance strategies [17], which undermine both sustained effort and progress toward goals.

Empirical research further indicates that this cognitive framing not only hinders task completion but also negatively impacts overall performance and well-being, as employees psychologically withdraw and reduce their investment in work [41]. Hence, hindrance appraisal provides a key explanatory mechanism by which SBLM undermines employee persistence and achievement, highlighting how leader behaviors indirectly shape workplace outcomes through appraisal processes. By examining this research, hindrance appraisals may play a significant role in how SBLM influences employees’ efforts to achieve their goals.

**Hypothesis 5b:** Employee hindrance appraisal mediates the relationship between supervisor bottom-line mentality and employee goal progress.

**Fig 1:**
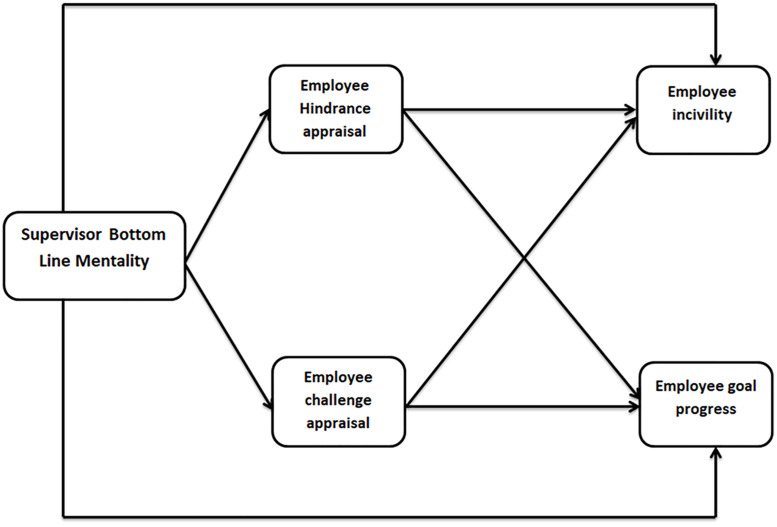
Research model.

The proposed research model for the hypothesized relations of supervisor bottom line mentality, appraisals and employee incivility as well as employee goal progress is presented in Fig 1.

## Materials and methods

### Population, sample and procedure

For this study, a self-administered paper questionnaire was used, a method commonly employed in research [23,44,45]. To mitigate potential common method bias, a unique time-lagged design was adopted. This design involved collecting data on both the independent and dependent variables at three distinct Time points, each separated by a two-week interval. This approach minimized the likelihood of respondents consistently answering or experiencing recall bias. In addition to the time-lagged design, other procedures were implemented to ensure validity, including participant anonymity, confidentiality, and clear instructions to minimize response bias.

Respect for participants’ privacy was a key consideration in this study. The questionnaire was accompanied by a cover letter that explained the study’s focus and requested voluntary participation from each respondent. Informed consent was clearly sought, and participants were guaranteed a high level of confidentiality regarding their responses, as the results would be used to generalize the responses to the entire study population. Their privacy concerns were addressed by clearly stating that the data would not be disclosed publicly, thereby ensuring the ethical conduct of the study.

The target population for this study consisted of full-time workers in the fast-moving consumer goods (FMCG) sector, specifically those involved in business-to-business sales within private and government firms. This sector was intentionally selected due to its performance-driven and highly competitive nature, providing a natural context for studying supervisor bottom-line mentality (SBLM). The frontline employees operate under continuous evaluation, with supervisors exercising considerable authority over customer allocations and performance feedback. Such structural dependence heightens the salience of supervisor bottom line orientation, making employees more sensitive to appraisals. Therefore, the fast paced nature of FMCG B2B sales requires constant self-regulation which aligns closely with the appraisal and coping pathways outlined in TTSC. Therefore the FMCG B2B context provides conditions under which the psychological effects of SBLM and mechanisms proposed in this study are likely to manifest. The interviewees were recruited through professional networks, LinkedIn groups, and organizational contacts in the FMCG sector.

The measuring instrument used for data collection was a three-level survey (A-I, B-II, and C-III) conducted at three Time points. Respondents rated their supervisors in terms of bottom-line mentality using Form A-I at Time 1 (T1), in which they completed the questionnaire by providing their demographic information. A unique employee ID was assigned to each participant to match the corresponding wave. Participants who completed Form A-I got Form B-II at Time 2 (T2), two weeks later. At the third Time (T3), participants who had already completed the previous two forms were given Form C-III, which evaluated their situation related to experiencing incivility and achieving objectives. Common method bias is also reduced since every survey was separated by two weeks.

Using Krejcie & Morgan’s (1970) [46] table, a sample size of 382 was determined based on a 95% confidence interval and a 5% margin of error. A total of 500 questionnaires were distributed, yielding 402 initial responses. However, responses from 20 employees were excluded because they were unmatched and incomplete, and the final sample comprised 382 employees (response rate: 76.4%). Convenience sampling was employed due to its practical advantages, such as ease of access and feasibility within the available timeframe. However, reliance on this non-probability technique represents a methodological limitation, as it restricts the representativeness of the sample and limits the generalizability of findings beyond the FMCG B2B sector studied.

Controls included demographic variables, such as age, gender, education, and tenure, in addition to the focal variables. To account for potential confounding effects and ensure the robustness of the results, the steps above were taken.

The data analysis was performed using Partial Least Squares Structural Equation Modeling (PLS-SEM). SmartPLS was chosen because of its suitability for predictive and theory-development studies, its ability to handle complex models with multiple latent variables, and its robustness in dealing with non-normal data distributions and smaller to medium sample sizes. Unlike covariance-based SEM, SmartPLS does not require strict normality assumptions and is particularly effective for testing mediation and moderation effects within models that emphasize both measurement and structural components. The non-parametric bootstrapping in PLS-SEM served to reliably test the hypothesized relationships rather than just making exploratory claims.

The data was collected in accordance with the institution’s ethical standards, and formal ethical approval was received from the Ethical Review Board of Capital University of Science and Technology, Islamabad, Pakistan (Ref: CUST/FMSS/REC/2025–53). The entire process of data collection adhered strictly to the established ethical guidelines for research involving human participants. Notably, this research did not include any experiments or clinical trials with humans or animals, nor did the questionnaire require any sensitive information.

### Instrumentation

The survey questionnaire was split into three sections. The first section contained the research purpose, accompanied by an assurance of confidentiality with the option for voluntary participation. The employee ID, age, gender, and professional experience were included in the second section. The research model variables appeared in the third section of the questionnaire, which included the following: supervisor bottom-line mentality (T1), challenge and hindrance appraisal (T2), and employee incivility and employee goal progress (T3).

The questionnaire utilized a five-point Likert scale that ranged from 1 (strongly disagree) to 5 (strongly agree). The existing four-item scale by [13] was modified for assessing supervisor bottom-line mentality. A sample item includes: “My supervisor is solely concerned with meeting the bottom line.” Hindrance and challenge appraisal were measured with eight items using the scale by [19]. Sample items include “It will help me to learn a lot” for challenge appraisal and “It will hinder any achievements that I might have” for hindrance appraisal. Employee incivility was measured with seven items developed by [25]. A sample item is: “I have put someone down or been condescending to others in my workplace.” Employee goal progress was evaluated through four items developed by [22]. A sample item is: “I have made considerable progress toward attaining this goal.”

### Pilot test

The pilot test, a significant phase in our research process, was conducted with 38 respondents to evaluate the clarity, validity, and reliability of the measurement instrument before full-scale data collection. The main objective was to ensure that all items were understandable, contextually appropriate, and capable of accurately capturing the constructs under study, thereby engaging our audience in the research process. Reliability was assessed using Cronbach’s Alpha, which measures internal consistency. A Cronbach’s Alpha value of 0.70 or above is generally considered acceptable for academic research [47].

Table 1 indicates results that all constructs exceeded the recommended threshold of 0.70, demonstrating acceptable internal consistency. Therefore, the instrument was deemed reliable and appropriate for further analysis.

**Table 1:** Reliability assessment of pilot test results.

Variable	No. of items	Cronbach’s Alpha
Challenge Appraisal	04	0.923
Employee Incivility	07	0.732
Goal Progress	04	0.839
Hindrance Appraisal	04	0.810
Supervisor Bottom Line Mentality	04	0.846

## Analysis and results

### Multicollinearity

In this research, the potential issue of multicollinearity was assessed using the Variance Inflation Factor (VIF) for each predictor variable. Multicollinearity occurs when independent variables are highly correlated, which can lead to inflated standard errors and unreliable regression coefficients. Following the conservative guideline suggested in previous literature, a VIF value below 5 is commonly considered acceptable [47], while some scholars suggest that values below 10 may be acceptable in less stringent contexts [48].

The VIF values obtained in this study were 1.600–1.904 for Challenge appraisal, 1.600–2.200 for Employee incivility, 1.600–1.700 for Goal progress, 1.645–1.771 for Hindrance appraisal, and 1.736–2.632 for Supervisor bottom line mentality. All values are well below the conservative cut-off of 5, indicating that multicollinearity is not a concern in this study

As per Table 2, results confirm that all values are well below the conservative threshold of 5, confirm that multicollinearity is not a concern in this dataset. This repeats the lack of concern about multicollinearity, providing a strong basis for the research validity and the stability of the relationships between the variables.

**Table 2:** Assessment of multicollinearity using Variance Inflation Factor (VIF).

Variable	VIF
Challenge appraisal 1	1.904
Challenge appraisal C2	1.600
Challenge appraisal C3	1.700
Challenge appraisal C4	1.600
Employee incivility 1	1.600
Employee incivility 2	1.700
Employee incivility 3	1.800
Employee incivility 4	2.000
Employee incivility 5	2.100
Employee incivility 6	2.200
Employee incivility 7	1.900
Goal progress 1	1.700
Goal progress G2	1.600
Goal progress G3	1.700
Goal progress G4	1.600
Hindrance appraisal 1	1.723
Hindrance appraisal 2	1.771
Hindrance appraisal 3	1.654
Hindrance appraisal 4	1.645
Supervisor bottom line mentality 1	1.736
Supervisor bottom line mentality 2	2.632
Supervisor bottom line mentality 3	2.157
Supervisor bottom line mentality 4	1.904

Note: VIF < 5 indicates acceptable multicollinearity; VIF ≥ 5 may indicate problematic multicollinearity.

There were multiple indicators which justified the choice of PLS-SEM to analyze the dataset. This method delivers optimum results when testing new relationships within research frameworks, especially for exploratory studies. Moreover, the prediction orientation of constructs becomes clear through its use. The out-sample prediction method developed by [47] serves as explanation for applying this method to the data analysis process.

### Measurement model

The research used variance based partial least squares structural equation modeling (PLS-SEM), as it is a nonparametric statistical approach [49]. The data quality was tested through checking maximum and minimum values against any entry errors. Moreover, kurtosis and skewness tests were run to determine if data is normally distributed. The results suggest that skewness and kurtosis met the required threshold of +−1 and +−2.

A measurement model served to evaluate the validity of the collected data. The composite reliability was run to check the reliability of the data which showed that all constructs exceeded the minimum required value of 0.70 [49]. The assessment of convergent validity utilized Average Variance Extracted (AVE) measurements which showed that the values were exceeding 0.50 for every construct. The measurement model retained the items that gave outer loadings below 0.70 because the values maintained the level above 0.50 [47].

To assess the discriminant validity, heterotrait-monotrait ratio of correlations (HTMT) was used [47]. The estimated HTMT values remained below the threshold of 0.90 hence validating discriminant validity among constructs. The results are displayed in Table 3.

**Table 3:** Measurement statistics for construct scales.

Constructs	OL	CR	AVE	α	VIF	MSV	ASV
**Supervisor Bottom-Line Mentality**		0.904	0.703	0.859	1.00	0.2480	0.1275
**SBLM1**	0.862						
**SBLM2**	0.883						
**SBLM3**	0.898						
**SBLM4**	0.878						
**Employee hindrance appraisal**		0.879	0.645	0.817	1.189	0.1275	0.0839
**HA1**	0.792						
**HA2**	0.818						
**HA3**	0.795						
**HA4**	0.807						
**Employee challenge appraisal**		0.932	0.775	0.903	1.00	0.0552	0.0253
**CA1**	0.825						
**CA2**	0.899						
**CA3**	0.882						
**CA4**	0.833						
**Employee Incivility**		0.954	0.747	0.943	1.157	0.2304	0.1069
**EI1**	0.862						
**EI2**	0.898						
**EI3**	0.902						
**EI4**	0.915						
**EI5**	0.877						
**EI6**	0.813						
**EI7**	0.774						
**Employee Goal Progress**		0.939	0.755	0.918	1.069	0.2480	0.1389
**GP1**	0.843						
**GP2**	0.904						
**GP3**	0.897						
**GP4**	0.905						

The evaluation of the measurement model led to the next step which focused on assessing the structural relationships among study variables. The structural model evaluation enabled to assess the structural associations between the variables.

### Structural model

The assessment of structural model included assessment through Effect size, out sample prediction, and coefficient of determination. The research used bootstrapping with 5000 resamples to compute t-values [49]. The research findings displayed that supervisor bottom line mentality (SBLM) had a strong and significant impact on hindrance appraisal (b = 0.357, p < 0.001) supporting H1a whereas SBLM also showed significant effect on challenge appraisal (b = 0.172, p < 0.001) thereby supporting H1b. SBLM positively influenced workplace incivility (INCIV) (b = 0.254, p < 0.001) while showing a negative impact on goal progress (GP) (b = −0.466, p < 0.001) thereby supporting H2 and H3 respectively.

The research findings showed that hindrance appraisal had negative effect on goal progress (b = −0.135, p < 0.001) while producing positive effect on incivility (b = 0.176, p < 0.001). The research results did not support the link between challenge appraisal and incivility as the results were not significant (b = 0.043, p > 0.05). Results also showed that challenge appraisal was positively related to goal progress. Table 4 displays the results obtained.

**Table 4:** Results of structural model analysis (hypotheses testing).

Relationships	β	SE	t-values	F2	R2
**SBLM → HIND APP**	0.357	0.0458	7.790	0.146	0.128
**SBLM → CHA APP**	0.172	0.0612	2.812	0.030	0.029
**SBLM → INCIVIL**	0.254	0.0490	5.185	0.065	0.136
**SBLM → GOAL**	−0.466	0.0430	10.919	0.257	0.276
**HIND APP → GOAL**	−0.135	0.0438	3.218	0.023	
**HIND APP → INCIVIC**	0.176	0.0480	3.667	0.030	
**CHA APP → INCIVIC**	0.043	0.0533	0.807	0.002	
**CHA APP → GOAL**	0.136	0.0539	2.469	0.023	

**Fig 2:**
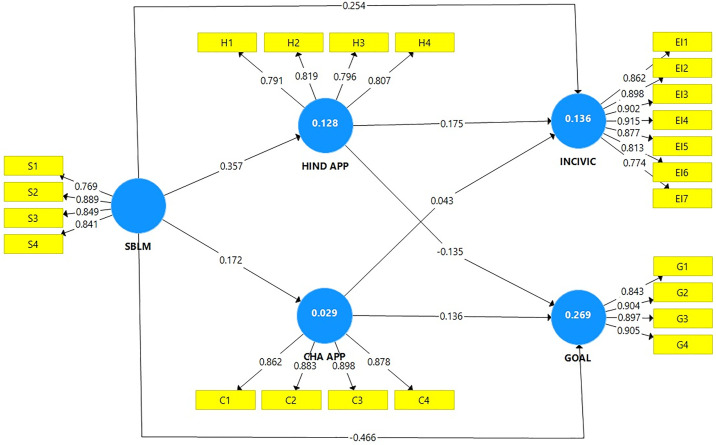
Structural model with standardized path estimates.

Beyond statistical significance, these coefficients also carry practical meaning. The effect of SBLM on incivility (β = 0.254) reflects a medium-sized impact, showing that supervisors’ strong bottom-line focus has a tangible and noticeable influence on workplace behaviors, elevating the risk of incivility in day-to-day interactions. The large negative coefficient of SBLM on goal progress (β = –0.466) highlights a particularly strong and practically meaningful effect, suggesting that prioritizing financial outcomes over other concerns can substantially hinder employees’ ability to achieve their goals. Even when the employees intend to perform well, the psychological strain caused by SBLM disrupts their ability to stay focused, plan effectively and sustain effort. The results further suggest that SBLM weakens employees’ capacity to convert goals into actual task accomplishment. In real workplace terms, this translates into slower task completion, reduced accuracy and diminished initiative. On the other hand, the positive coefficient for challenge appraisal on goal progress (β = 0.136) indicates a smaller but still meaningful effect size, illustrating that when employees interpret bottom-line pressure as a challenge, it can provide a motivational boost to their performance. In this way, the results underscore that SBLM is not just statistically significant but substantively important in shaping workplace outcomes. Fig 2 shows structural model with standardized path coefficients, outer loadings, and R² Values.

### Bootstrapping for testing mediation effects

As depicted in Table 5, the sequential mediation model tested four mediation pathways. Each pathway achieved its statistical significance by utilizing 95% confidence interval. A mediation relationship existed when the zero value fell outside the established confidence interval bounds indicating strong evidence of mediation. The testing of SBLM to INCIV via HA found statistically significant results (b = 0.063, CI = 0.029 ~ 0.104) thereby supporting hypothesis H4a. The pathway from SBLM to GP through CA demonstrated a significant mediation effect (b = 0.023, CI = 0.002 ~ 0.050) confirming the study’s robust findings and supporting hypothesis 4b. The relationship between SBLM and GP through HA was found statistically significant (b = −0.048, CI = −0.089 ~ −0.019) thus supporting hypothesis 5b.

**Table 5:** Bootstrapping test 95% CI (standardized).

Paths	Standardized coefficient (ß)	Bootstrapping 95% CI	
Direct effects		Lower limit	Upper limit
**SBLM to HA**	0.357	0.272	0.444
**HA to EI**	0.175	0.087	0.271
**CA to GP**	0.136	0.030	0.240
**SBLM to CA**	0.172	0.042	0.271
**CA to EI**	0.043	−0.062	0.140
**Indirect effects**			
**SBLM → HA → EI**	0.063	0.029	0.104
**SBLM→ HA → GP**	−0.048	−0.089	−0.019
**SBLM→ CA → GP**	0.023	0.002	0.05
**SBLM→ CA → EI**	0.007	−0.010	0.028

SBLM = Supervisor Bottom Line Mentality, HA = Hindrance Appraisal, CA = Challenge Appraisal, GP = Goal Progress, EI = Employee Incivility.

Results confirmed the sequential mediating effect of supervisor bottom line mentality on the relationship between employees’ appraisal of the mentality and employee outcomes. However, the pathway from SBLM to EI via CA (β = 0.007, CI: −0.010 ~ 0.028) showed insignificant mediating effects which confirmed that viewing supervisor’s bottom line mentality as a challenge does not reduce or increase uncivil behavior. Therefore, hypothesis H5a is rejected.

These mediation findings reinforce the central insight: employees’ appraisal processes are pivotal in explaining why the same supervisory bottom-line focus can generate either adaptive or maladaptive outcomes. Hindrance appraisals amplify negative behaviors such as incivility, while challenge appraisals provide a weaker but positive channel toward goal achievement. This directly aligns with the Transactional Theory of Stress and Coping (TTSC), which emphasizes appraisal as the mechanism through which stressors shape outcomes. By embedding SBLM into this framework, the results demonstrate that leadership’s bottom-line focus does not exert uniform effects but operates through employees’ subjective interpretations to produce divergent behavioral consequences.

## Discussion

The purpose of this study was to investigate the impact of supervisor bottom line mentality on the employee outcomes through appraisal of the employees. It examines the variables using SmartPLS-SEM. Data from 382 employees of FMCG B2B sector supports the model’s accuracy in predicting employees’ career success with the appraisals playing a critical role regarding the bottom-line mentality.

The results confirm that supervisors’ bottom line mentality leads to incivility. This finding aligns with and extends previous research highlighting the unfavorable consequences of BLM in organizational contexts [13]. Prior research shows that when leaders only focus on results, they can unintentionally create a toxic environment where mistreatment becomes more common. Working under such supervisors creates strain, which causes employees to act uncivilly [17,50].

Furthermore, it was found that appraisals play a significant role in shaping employees’ responses towards supervisors’ bottom line mentality. Previous literature has supported this finding suggesting that employees’ appraisals of their leader’s behaviors shape their behavioral responses [51]. This finding also aligns with prior research (e.g., [52] a single stressor may simultaneously elicit evaluations of challenge and hindrance, contingent on the situation and the individual’s perception [42]. The dynamic and subjective character of stress assessment procedures is reflected in this nuanced viewpoint, which has significant implications for understanding workplace behaviors and outcomes. This reflects the dynamic nature of stress appraisal process, where the line between productive pressure and destructive overload is blurred [53]. Leaders must not only consider the demands they impose, but also how these demands are interpreted in their teams [54]. Recognizing this distinction is critical for planning interventions that reduce strain while maintaining engagement.

Although the present model is theoretically sound, there are numerous alternative interpretations that can be proposed to explain the findings. As another example, organizational culture can support bottom-line-focused supervision while also allowing for an escalation of tolerance towards incivility, making it challenging to capture the impact of SBLM alone [55]. Secondarily, individual differences in employee personality, such as neuroticism or trait aggression, may also moderate the appraisal of SBLM and its conversion into incivility [56]. These relationships can also be moderated by job characteristics, such as role clarity and autonomy [57]. Lastly, since stress appraisals can change over time, longitudinal studies can help reveal any changes where a challenge that was initially motivating becomes a perceived obstacle as resources are exhausted.

A particularly notable and unexpected finding was the rejection of H5a: the pathway from SBLM to incivility through challenge appraisal was not significant. This suggests that even when employees view bottom-line demands as developmental opportunities, such appraisals do not reduce (or exacerbate) incivility. This has significant implications for organizational behavior, as it indicates that challenge appraisals, while effective in energizing employees towards task performance, do not extend to regulating relational conduct. This aligns with research showing that challenge appraisals are more strongly associated with task-focused outcomes than with social behaviors such as civility [58]. In high-pressure environments, employees may channel their motivational energy into achieving performance goals, while relational behaviors remain unaffected [59].

Cultural and contextual influences may also explain this finding. In the Pakistani FMCG B2B sector, competition and hierarchical structures may normalize abrasive interactions, reducing the protective effects of challenge appraisals on civility. Research on organizational culture in Pakistan highlights substantial power distance and collectivist norms that can legitimize more rigid interpersonal dynamics under performance pressure [60]. Employees may perceive incivility as an acceptable or even necessary strategy for coping with pressure and achieving results. In such contexts, challenge appraisal strengthens motivation but does not constrain counterproductive relational behaviors. This research opens up avenues for future studies to test whether this pattern persists in collectivist cultures that emphasize relational harmony, or in sectors with lower performance pressure, thereby contributing to a deeper understanding of organizational behavior and cultural influences.

The results further stated that even if the employees appraise the bottom-line mentality of their supervisors as a challenge, the possibility of them engaging in uncivil behavior does not increase nor decrease. Therefore, even if bottom line behaviors are perceived as positive, they might not be enough to control negative behaviors in stressful work environments [53].

This insignificant mediation has theoretical implications. It suggests that the TTSC framework needs to be applied more carefully when linking appraisals to different outcome domains. Challenge appraisals appear to direct energy toward personal accomplishment and goal progress, but not toward relational outcomes such as civility. This boundary condition underscores the importance of integrating appraisal theory with complementary perspectives, such as moral disengagement theory, which explains why employees may justify uncivil acts as acceptable in the pursuit of success [40]. Future research should also consider curvilinear and interaction effects, as challenge stressors may only influence incivility at very high or prolonged levels, which were not captured in the current model [61]. Although this result initially appears counterintuitive within the TTSC framework, it can be better understood by considering additional psychological mechanisms that shape how employees rationalize behavior under pressure. This phenomenon can further be understood through moral disengagement theory which explains how individuals rationalize unethical or counterproductive behavior when pursuing valued goals. When supervisors emphasize bottom line outcomes, employees who perceive these demands as challenges may prioritize goal attainment so strongly that they cognitively disengage from moral self-regulation, justifying minor acts of incivility as necessary tradeoffs for success. Thus, even when challenge appraisals energize employees towards performance, they may lower their sensitivity to interpersonal norms.

Lastly, the findings suggest that supervisors need to be pay careful attention to the fact that hindrance appraisal can lead to more negative psychological pathways characterized by feelings of obstruction, or resource inadequacy, resulting in employee incivility which hampers organizational performance [42]. Therefore, supervisors must be mindful of how their expectations are perceived, which can help in preventing the spillover of strain into incivility or other counterproductive behaviors.

### Implications

Practically, there are specific interventions that organizations should consider. The significance of performance pressure, in the manner in which it is conveyed and received, ought to be emphasized in supervisory training [62]. The leadership training should move beyond generic goal setting modules to include specific workshops on ethical decision making and emotional intelligence. These modules can help managers in recognizing when the performance pressure begins to create psychological strain among employees and how to redirect it through supportive behaviors.

Organizations can also adopt such feedback mechanisms where employees anonymously rate their supervisors on relational and ethical dimensions. Such systems can help organizations identify early warning signs of the bottom line focus and provide targeted mentoring interventions.

The anonymous perception surveys enable organizations to track how SBLM is being evaluated [63]. It has also been suggested that providing sufficient resources, such as support systems and role clarity, may enable employees to construe supervisor pressure as a challenge rather than an impediment [37]. Lastly, performance management systems should be revised. The addition of interpersonal behaviors including civility, engagement and feedback quality to KPIs would help reverse the trend of disregarding civility under pressure as well as reinforce that how results are achieved is as important as the results themselves. However, the current findings indicate that resource provision and positive framing alone may not guarantee civility; explicit emphasis on interpersonal norms (e.g., embedding civility into KPIs, leader evaluations, and team-level expectations) is also required to prevent relational harm in high-pressure environments.

### Limitations and future directions

This study provides insight into how employee attributions of supervisor bottom line mentality impact the employees’ outcomes in Pakistan. The transactional theory of stress and coping offers a robust theoretical framework to understand these dynamics, however future studies could investigate how organizational culture, leadership style difference and perceived organizational support influence these relationships.

Although the current study employed a time-lagged survey design with three-time points, it is essential to note that the mediating variables (i.e., challenge and hindrance appraisals) were not assessed repeatedly over time. Therefore, the research is not able to draw causal conclusions regarding mediation effects. The mediators are cross-sectional, which restricts the capacity to establish the temporal sequencing among the variables needed to affirm causality. Longitudinal studies with repeated measures or diary designs should be used in the future to confirm the directional links in the suggested model.

While this study focused on understanding how appraisal of employees regarding supervisor bottom line mentality affects employee outcomes, numerous limitations should be noted. Although this study employed a time lag, collecting responses from employees during different periods to reduce the likelihood of single-source bias, it still could not be considered a longitudinal study. It is suggested that future studies replicate the model using a longitudinal research design.

Although the study was conducted in FMCG B2B sector which could limit the generalizability of the findings, future studies could apply the conceptual model to other sectors for a thorough comprehension. In particular, the bottom-line mentality of supervisors may reveal various contextual meanings across different fields, including healthcare, IT, public service, and education. In addition, the cross-cultural generality of the appraisal mechanisms identified in this study can be further determined through wider geographic replication (i.e., research conducted in Western, East Asian, or African cultural settings).

The current study aimed on the supervisor bottom-line mentality from the perspective of the employee, whereas it is possible that the supervisor may have different intentions behind their bottom-line focus, which were not captured in this research. Therefore, future studies can examine the supervisors’ bottom line mentality from both the supervisor and employee perspective, allowing for a more comparative analysis of the differences among their viewpoints. To grasp these subtle perceptions and opposing thoughts, future research should consider a mixed-methods design or the application of a qualitative research method, such as interviews or narrative diaries. Such approaches would aid in revealing situational clues, affective polarity, and leader rationale that may be overlooked in closed-ended survey tools. This would add value to the knowledge of appraisal procedures and enhance the perception of leadership behavior interpretations.

Since this study employed convenience sampling, the findings may not be generalizable to a wider population. Lastly, this research employed a quantitative research approach which allowed for testing of hypotheses. Future research could incorporate qualitative methodology to enrich the findings and provide a deeper understanding of the phenomenon under study by depicting the contextual factors and complex dynamics that may not emerge through quantitative measures alone.

## Supporting information

S1 File Data set. (CSV)

S2 File Questionnaire. (PDF)
