# Supplementary material for: Supervisor bottom line mentality and its impact on employee outcomes: The mediating role of employee appraisals
Source: PLoS One. 2026 Jan 20;21(1):e0338024. doi: 10.1371/journal.pone.0338024 (PMC12818646; doi:10.1371/journal.pone.0338024)
Supplement: S2 File — (PDF) [file pone.0338024.s002.pdf]

Supervisor Bottom Line Mentality

Rate the following statements, while keeping in view your supervisor.

**1 = Strongly Disagree- 2 = Disagree- 3 = Neutral- 4 = Agree- 5 = Strongly Agree**					
My supervisor is solely concerned with achieving sales targets.	**1**	**2**	**3**	**4**	**5**
My supervisor only cares about the business.	**1**	**2**	**3**	**4**	**5**
My supervisor treats achieving sales targets as more important than anything else.	**1**	**2**	**3**	**4**	**5**
My supervisor is more concerned about his/her interests than the happiness of employees.	**1**	**2**	**3**	**4**	**5**

Challenge and Hindrance Appraisal

Rate the following statements, while keeping in view your supervisor.

**1 = Strongly Disagree- 2 = Disagree- 3 = Neutral- 4 = Agree- 5 = Strongly Agree**					
**Challenge appraisal**					
My supervisor's sole focus on achieving sales targets helps me to learn a lot	**1**	**2**	**3**	**4**	**5**
My supervisor's sole focus on achieving sales targets makes the experience educational	**1**	**2**	**3**	**4**	**5**
My supervisor's sole focus on achieving sales targets shows me I can do something new	**1**	**2**	**3**	**4**	**5**
My supervisor's sole focus on achieving sales targets keeps me focused on doing well	**1**	**2**	**3**	**4**	**5**

**Hindrance appraisal**					
My supervisor's sole focus on achieving sales targets hinders any achievements I might have	**1**	**2**	**3**	**4**	**5**
My supervisor's sole focus on achieving sales targets restricts my capabilities	**1**	**2**	**3**	**4**	**5**
My supervisor's sole focus on achieving sales targets limits how well I can do	**1**	**2**	**3**	**4**	**5**
My supervisor's sole focus on achieving sales targets prevents me from mastering difficult aspects of the work	**1**	**2**	**3**	**4**	**5**

Employee Incivility

Please judge how each statement fits your behavior.

**1 = Never — 2 = Rarely — 3 = Once in a while — 4 = Often — 5 = Frequently**					
I have put someone down or been condescending to others in my workplace.	**1**	**2**	**3**	**4**	**5**
I have paid little attention to someone’s statement or showed little interest in their opinion.	**1**	**2**	**3**	**4**	**5**
I have made demeaning or derogatory remarks about someone in my office.	**1**	**2**	**3**	**4**	**5**
I have addressed a colleague in unprofessional terms, either publicly or privately.	**1**	**2**	**3**	**4**	**5**
I have ignored or excluded a colleague from professional camaraderie.	**1**	**2**	**3**	**4**	**5**
I have doubted a colleague’s judgment on a matter over which they had responsibility.	**1**	**2**	**3**	**4**	**5**
I have made unwanted attempts to draw a colleague into a discussion of personal matters.	**1**	**2**	**3**	**4**	**5**

Goal Progress

Indicate the extent of your agreement or disagreement with the below statements.

**1 = Strongly Disagree- 2 = Disagree- 3 = Somewhat Disagree- 4 = Neutral- 5 = Somewhat Agree**					
I am making good progress toward achieving my goals.	**1**	**2**	**3**	**4**	**5**
I am on track to accomplish my goals.	**1**	**2**	**3**	**4**	**5**
I feel satisfied with the progress I have made toward my goals.	**1**	**2**	**3**	**4**	**5**
I am achieving the milestones I have set for my goals.	**1**	**2**	**3**	**4**	**5**
